# Image features for quality analysis of thick blood smears employed in malaria diagnosis

**DOI:** 10.1186/s12936-022-04064-2

**Published:** 2022-03-05

**Authors:** W. M. Fong Amaris, Carol Martinez, Liliana J. Cortés-Cortés, Daniel R. Suárez

**Affiliations:** 1 Programa de Doutorado em Biotecnologia, Universidade Federal do Pará, Belém, Brazil; 2 SnT-Interdisciplinary Centre for Security, Reliability and Trust, University of Luxembourg, 29, avenue John F. Kennedy, 1855 Luxembourg Kirchberg, Luxembourg; 3 Laboratory of Parasitology, National Health Institute of Colombia, Bogotá, Colombia; 4 School of Engineering, Pontificia Universidad Javeriana, Bogotá, Colombia

**Keywords:** Image processing, Coloration quality, Malaria diagnosis, TBS

## Abstract

**Background:**

The World Health Organization (WHO) provides protocols for the diagnosis of malaria. One of them is related to the staining process of blood samples to guarantee the correct parasite visualization. Ensuring the quality of the staining procedure on thick blood smears (TBS) is a difficult task, especially in rural centres, where there are factors that can affect the smear quality (e.g. types of reagents employed, place of sample preparation, among others). This work presents an analysis of an image-based approach to evaluate the coloration quality of the staining process of TBS used for malaria diagnosis.

**Methods:**

According to the WHO, there are different coloration quality descriptors of smears. Among those, the background colour is one of the best indicators of how well the staining process was conducted. An image database with 420 images (corresponding to 42 TBS samples) was created for analysing and testing image-based algorithms to detect the quality of the coloration of TBS. Background segmentation techniques were explored (based on RGB and HSV colour spaces) to separate the background and foreground (leukocytes, platelets, parasites) information. Then, different features (PCA, correlation, Histograms, variance) were explored as image criteria of coloration quality on the extracted background information; and evaluated according to their capability to classify images as with Good or Bad coloration quality from TBS.

**Results:**

For background segmentation, a thresholding-based approach in the SV components of the HSV colour space was selected. It provided robustness separating the background information independently of its coloration quality. On the other hand, as image criteria of coloration quality, among the 19 feature vectors explored, the best one corresponds to the 15-bins histogram of the Hue component with classification rates of > 97%.

**Conclusions:**

An analysis of an image-based approach to describe the coloration quality of TBS was presented. It was demonstrated that if a robust background segmentation is conducted, the histogram of the H component from the HSV colour space is the best feature vector to discriminate the coloration quality of the smears. These results are the baseline for automating the estimation of the coloration quality, which has not been studied before, but that can be crucial for automating TBS’s analysis for assisting malaria diagnosis process.

## Background

Malaria is an infectious disease and one of the most important public health problems worldwide. Five parasites species cause human malaria from the *Plasmodium* genus, namely *Plasmodium vivax*, *Plasmodium falciparum*, *Plasmodium malariae*, *Plasmodium ovale* and *Plasmodium knowlesi.*. According to the World Health Organization (WHO), in 2019, 228 million malaria cases were estimated worldwide. In most countries where malaria is endemic, the transmission occurs in rural areas where the coverage of the laboratory network is weak or does not exist [1].

Currently, there are two main microscopic methods of malaria: analysis of thick blood smears or thin blood smears. The analysis of thick blood smears (TBS) is the reference method chosen as a first option for the malaria diagnosis worldwide [2]. In this method, after a blood sample is taken and dried, the TBS is stained. In Colombia, the type of stain used to stain the samples is modified Romanowsky. It is a stain that does not include in its composition alcohol-derivative substances like methanol, it does not precipitate easily, in addition to being an inexpensive good quality option, recommended by the WHO and INS [3–6]. After the staining process, the smear is visually analysed by microscopy, where parasites and leukocytes are identified and counted.

For the malaria diagnosis process, the WHO provides protocols for obtaining the blood sample, conducting its staining process, estimating the parasitaemia, and protocols for the storage of the analysed sample [1, 7]. In published reports, the WHO has emphasized the need for all laboratories responsible for malaria diagnosis to fully comply with a strict inspection of the diagnostic techniques they implement in order to guarantee the correct diagnosis of the disease [3]. However, in rural diagnostic centres the attention coverage of the laboratory network is weak or even non-existent [8], making it challenging to fulfil the recommendations given by the WHO according to the sample management, the staining, and diagnosis procedures. When a smear is not stained correctly, it is difficult to identify the parasites visually.

The quality of the thick blood smears can be affected by the following factors: the quality of the stain, the time and storage place of the stain, the stain concentration, the pH of both the staining solution and the water used for rinsing the smear. Indirectly, factors such as the limited availability of materials, the conditions and place of sample preparation, and the number of patients to attend (not enough personnel to guarantee the time required for staining the smears) can affect the staining procedure. The previously mentioned factors are common in rural diagnostic centres, were patients travel a long distance to receive diagnosis [6, 8–10].

Image processing has been used throughout the years for parasite diagnosis and classification. However, to the authors’knowledge, image processing has not yet been used by the research community to evaluate the staining procedure of the smear. The latter is crucial to ensure the visualization of the parasite by microscopy. Computer vision techniques applied to parasite detection and classification in blood smears have been used in previous studies as a response to the need for new alternatives to reach the reduction of malaria for 2030 [11]. Images features such as colour, texture, shape, and size combined with machine learning techniques are commonly used for parasite detection. For example, Hanif et al. [12] and Salamah et al. [13] used features based on histograms for malaria parasite detection. Those studies used thresholding-based segmentation and colour-based histograms, respectively.

Coloration quality analysis in thick blood smears employed in malaria diagnosis is an unexplored field. It requires attention because parasite identification and counting can be affected by the smear’s coloration. Taking into account the influence of the staining procedure in all the malaria diagnosis process, the purpose of this work is to analyse an image-based approach to describe the coloration quality of thick blood smears stained with the Romanowsky stain. The coloration analysis can be useful to indirectly evaluate the quality and reliability of the diagnostic results.

In this work, it is taken into account the biological criteria that define quality in thick blood smears, and it is explored different algorithms to find image-based criteria to defined the coloration quality of the smear.

## Methods

**Fig. 1:**
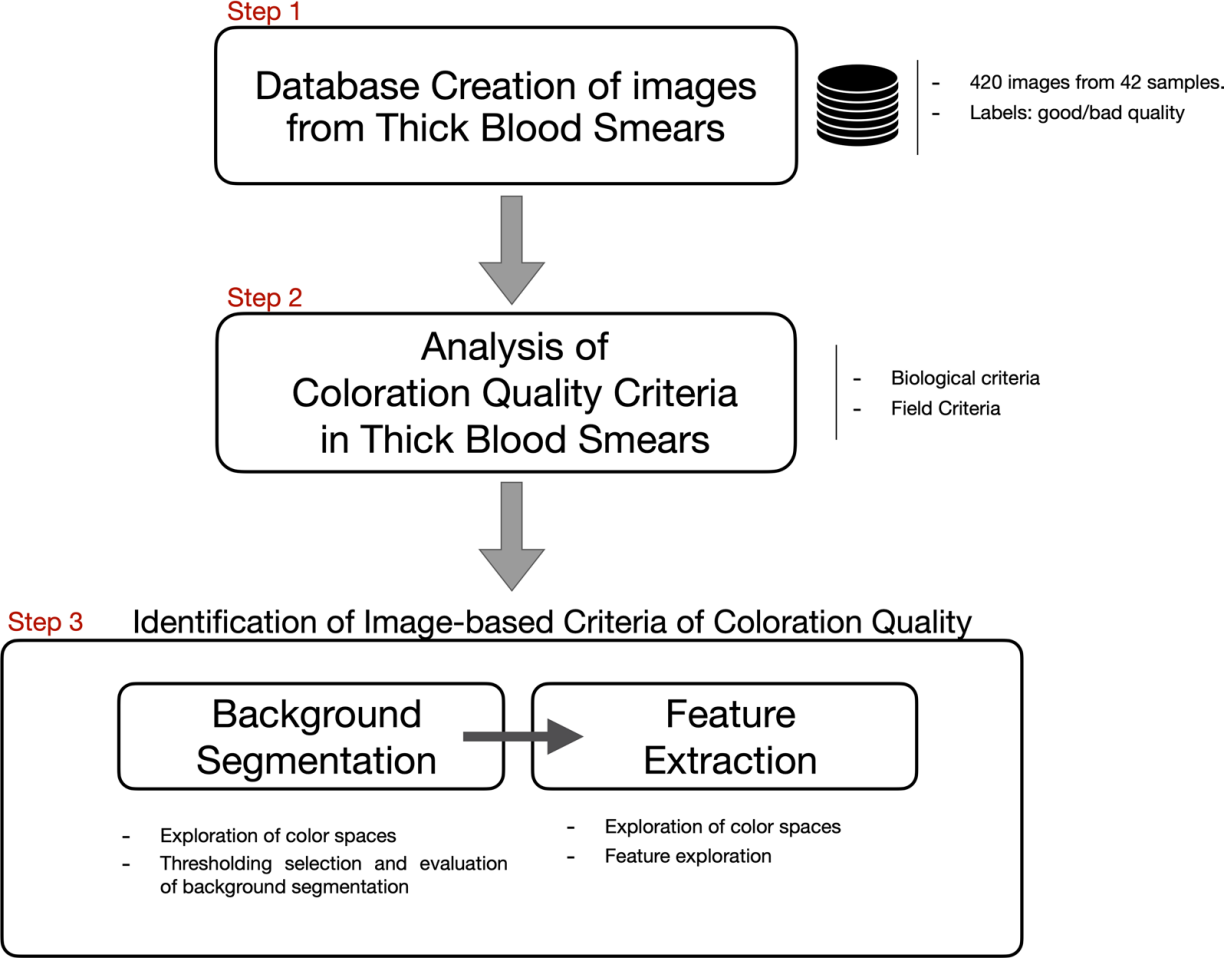
Methodological steps followed to investigate the coloration quality in thick blood smears used for malaria diagnosis

The three-steps methodology presented in Fig. 1 was followed to investigate an image-based approach to evaluate the coloration quality of the staining process in thick blood smears (stained with the modified Romanowsky stain). The first step corresponds to creating a database with images of thick blood smears with good and bad coloration quality. This database allows the exploration and evaluation of different image-based approaches. The second step corresponds to the analysis of existing coloration quality criteria used in laboratories for evaluating the quality of thick blood smears (including analysis of the background of the sample, analysis of the color of the leukocytes, shape of the cytoplasm). This step allows finding which of those criteria could be used to identify the coloration quality using only image data. Finally, the third step corresponds to identifying image-based criteria to evaluate the quality of the coloration. Different image-based processing techniques were tested to finally select the most suitable features to identify the coloration quality.

### Database creation

The image database was created to design and evaluate different image-based strategies that help determine the quality of the staining procedure of thick blood smears. Currently, there are no available studies in the literature that use databases with images from thick blood smears stained with Romanowsky stain. Most of the published papers have studied thick blood smears for parasite detection using the Giemsa stain [14–17] and Jaswant Singh Battacharya stain [18]. In those cases, the database has not been offered for the public domain and, in most of them, the number of images used is low (e.g. 75 – 256 images, except for the work published by Yang et al. [15] where the database consisted in a total of 1819 images).

The control of the image acquisition process was an essential factor in guaranteeing the repeatability of this study. In addition to the coloration variability in an image due to the blood smear preparation process mentioned above, factors such as the amount of light passing through the microscope’s eyepieces, the lens, the camera, and the microscope types can affect the colour information of the image.

**Fig. 2:**
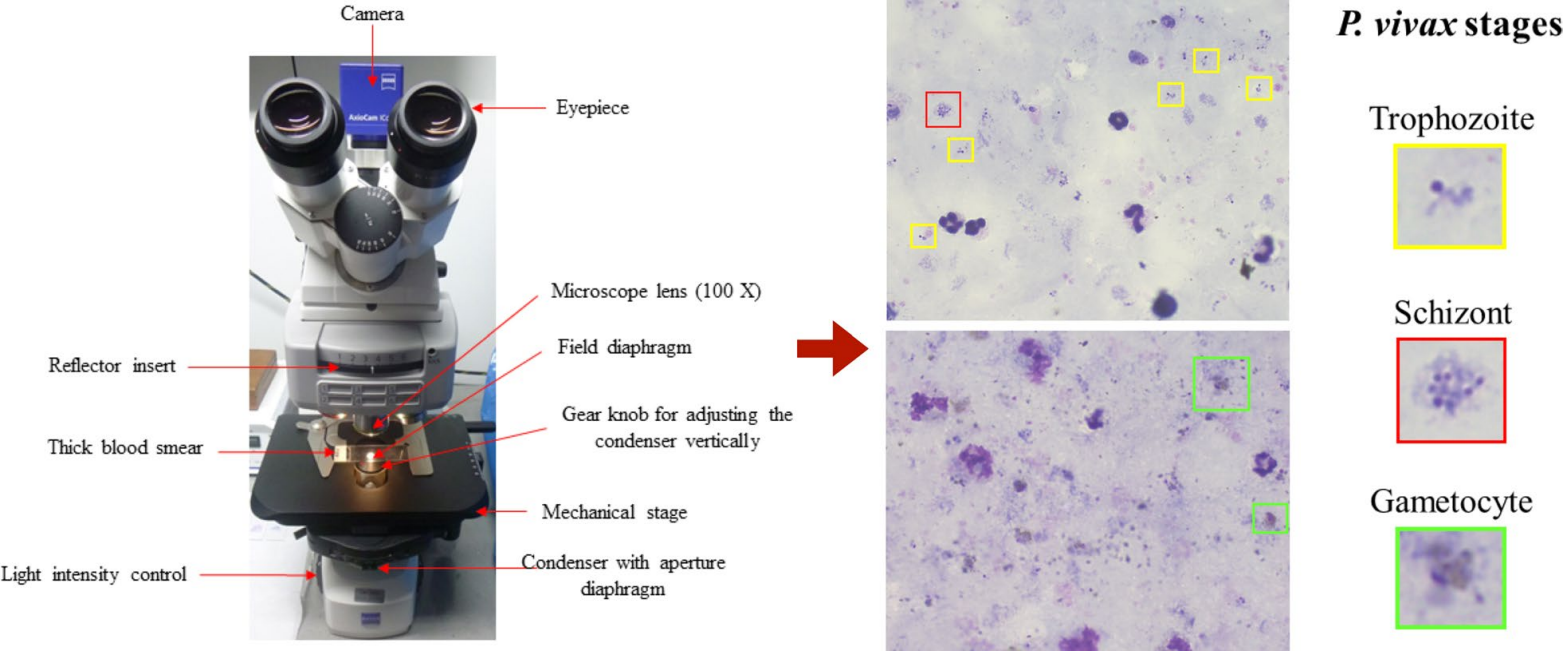
An optical microscope used to create the dataset of thick blood smears. It is an Axio Zeiss Scope A1. The red arrows indicate the microscope parts that were important for creating the dataset [8]. Images on the right correspond to microscopic images of thick blood smear with good coloration quality. Colour boxes show the life cycle stages of the parasite *P. vivax* as seen in the microscope. The yellow square shows the trophozoite; red, the schizont; and green, the gametocyte stage. Resolution: 1000X. Reference: Author

Because of this, the images were captured following specific parameters to en- sure that the acquired images contained the same colour information as the one seen through the microscope. The LED-Illuminated optical microscope (Axio Zeiss Scope.A1, Carl Zeiss, Germany) showed in Fig. 2 was used to create the database. Because it is a LED-Illuminated microscope, the blue filter between the light source and the condenser was not required. The figure also shows the 100X microscope lens used for the microscopic visualization (Reference: Objective-lens A-Plan 100x/1,25 Oil M27, Brand: Carl Zeiss, Country: Germany) and examples of microscopic images of thick blood smears.

The amount of adjustments that the microscopist has to make to the microscope’s illumination is a variable that can be related to the background coloration quality in blood smears. Depending on the thickness and the amount of stain, a stained blood smear can require a significant quantity of light to visualize the sample. For the image dataset it was controlled several elements from the microscope to guarantee similar lighting conditions during the images acquisition:

- Reflector insert: this element was kept in the centre to avoid modifications in the light conditions.
- Field diaphragm: this element was kept in its maximum opening point.
- Gear knob for adjusting the condenser vertically: this element was kept as close to the thick blood smear as possible.
- Condenser with aperture diaphragm: this element was kept in the centre to avoid modifications in the light conditions.
- Light intensity control: this element was used to standardize the light quantity through the eyepieces. The amount of light was established using a light metre (Model 407026, Extech$^{TM}$, USA). An average of 22,4 lux was the defined amount of light. This value was established according to the recommendations given by professionals in charge of malaria diagnosis at the National Health Institute of Colombia (INS).

The study population corresponds to cases of malaria due to *P. vivax* during the years 2017 and 2018. *Plasmodium vivax* is the predominant parasite in the WHO Region of the Americas, representing 75% of malaria cases [1]. A complete set of 42 thick blood smears used for malaria diagnosis were used (Not all the samples are infected with malaria parasite). It was obtained 420 photographic records (10 images of different fields per slide) from those samples. The fields captured correspond to fields located close to the centre of the sample to ensure uniformity in the images (in periphery regions, the blood thickness can vary). From the 420 images, 210 correspond to images with good coloration quality and 210 with bad coloration quality (balanced classes). The thick blood smears classification (good or bad quality) was defined based on the background colour of the sample when visualizing it with the microscope and according to the coloration description defined by the INS-Colombia [8] that follows the one provided by the WHO [2, 4] and also used in other works [19, 20].

If the background showed as a pink or light blue colour with pink areas in the microscopic field, it was considered of bad quality. Otherwise, the image was classified with good coloration quality.

Different factors affect the quality of the coloration of the thick blood smear: errors in pH of the staining solution, errors in the time of coloration, and the thickness of the sample.

- Smear’s background pink. This colour could be because the pH of the staining solution is acid, or the coloration time was not enough, or the thickness of the smear was light. With this type of background, it is most probable to get a poor differentiation between the chromatin of parasites and the artefacts present in the sample. The pink background does not allow the identification of biological elements as quickly as the blue background. For example, parasite’s chromatin and circular artefacts may look very similar to each other without the proper background.
- Background blue. It is less probable that the pH of the solution is poorly prepared as in pink smears. With a blue background and a LED-light type (as used in this research), it is easier to see and distinguish the parasites from other particles (like artefacts) present in the sample.

The images were labelled by personnel certified in the identification of the malaria parasite stages (one of the authors: WM Fong Amaris) using the web-based labelling tool named Labelbox [21]. The images acquired through the optical microscope have 2056 x 2452 pixels and were saved in PNG (Portable Network Graphics) format.

### Coloration quality criteria

Depending on the quality of the staining procedure, i.e. depending on the resulting coloration quality of the thick blood smear, the parasites can be easily detected or not. Different factors indicate how good the coloration quality is, such as:

- Background colour of the smear.
- Colour of the nucleus and the cytoplasm of the leukocytes and parasites perceived with the microscope.
- Colour and morphology of the platelets.
- Small differences in pH.

Figure 2 shows two microscopic images that correspond to thick blood smears with good coloration quality, where the parasites can be easily identified [8]. From this figure, it is possible to see examples of the life cycle stages of the parasite *Plasmodium vivax* (yellow and red squares correspond to the trophozoite and schizont stages, respectively. Green square represents the gametocyte stage).

#### Biological criteria

Standard criteria defined by the WHO and national health institutions to define the quality of the thick blood smear samples were analysed and compared with the criteria used in field laboratories. The analysis was conducted to define the critical quality criterion that can be used to automate the process of determining the quality of thick blood smear samples using only image data.

- Standard criteria
  The WHO [2], the INS-Colombia (National Health Institute) [8], Field [19], and López [20], have reported coloration criteria that should be complied with to guarantee an effective malaria diagnosis. The following list corresponds to the elements that can be visualized in a microscopic field from thick blood smears and their expected appearance if the staining process was carefully followed.
  - Smear background: pale blue
  - Platelets: Intense pink to violet with stippling
  - Leukocytes: pale pink cytoplasm and blue nucleus. Approximately 10–20 leukocytes per microscopic field. Depending on the leukocyte, it will present granulation.
  - Nucleus or chromatin: Red or violet chromatin dots with attached blue cytoplasm or presence of one chromatin dot attached to a ring of blue cytoplasm that can or cannot contain a vacuole.
  - Cytoplasm: Irregular or fragmented blue cytoplasm.
  - Pigment: Pale yellow or intense brown.
  Additionally, Table 1 shows an example of the expected colour in some of the elements listed before.
- Field criteria
  In field laboratories in charge of malaria diagnosis, some of the standard criteria described above are the ones used as indicators of the quality of the sample. The main factors used are: the non-visualization of erythrocytes in the background of the sample, the blue background color, the chromatin colour of the parasite (it has to be red or purple-red) and, nucleus colour from the leukocytes that it must be blue or purple [2, 8].
  The analysis conducted to criteria followed by field laboratories indicates that the appearance of the smear’s background is one of the most determinant features of the smear’s quality aside from the biological features. However, it is important to mention that in practice, the absolute and determinant judgment about the coloration quality of the stained sample is only declared after its microscopic visualization. Therefore, a bad coloration quality could affect the malaria diagnosis process by adding confusion to microscopists during the parasite identification process.

**Table 1:**
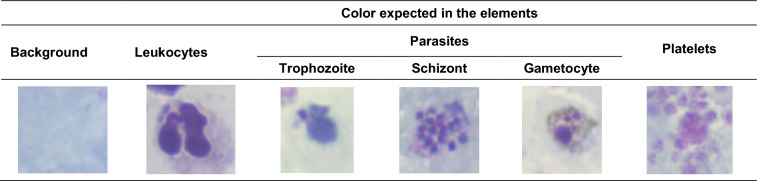
Colour appearance of biological elements after the staining procedure of thick blood smears. Table based on the recommendation of WHO [2] and the INS-Colombia [8]

### Image-based criteria of quality: background segmentation

As mentioned before, a poor staining procedure affects the quality of the blood smear and, therefore, will impact the malaria diagnosis process. This paper explores different image-based features to automatically estimate the coloration quality from microscopic images of thick blood smear. The MATLAB image processing toolbox was used to conduct the analysis [22].

Both the biological criteria of quality and the criteria used in field laboratories showed that the appearance of the smear background is a key feature to evaluate the quality of the coloration. Therefore, from the microscopic images of the TBS, the background information was separated from the foreground information (leukocytes, parasites, and platelets) to ensure that the image-based analysis is conducted only on the background information.

To achieve this, a segmentation procedure in different colour spaces based on manual image thresholding, was analysed. Different components of the RGB and HSV colour spaces were used, based on previous results obtained in the literature in the area of image-based malaria diagnosis [15–18, 23–29].

**Fig. 3:**
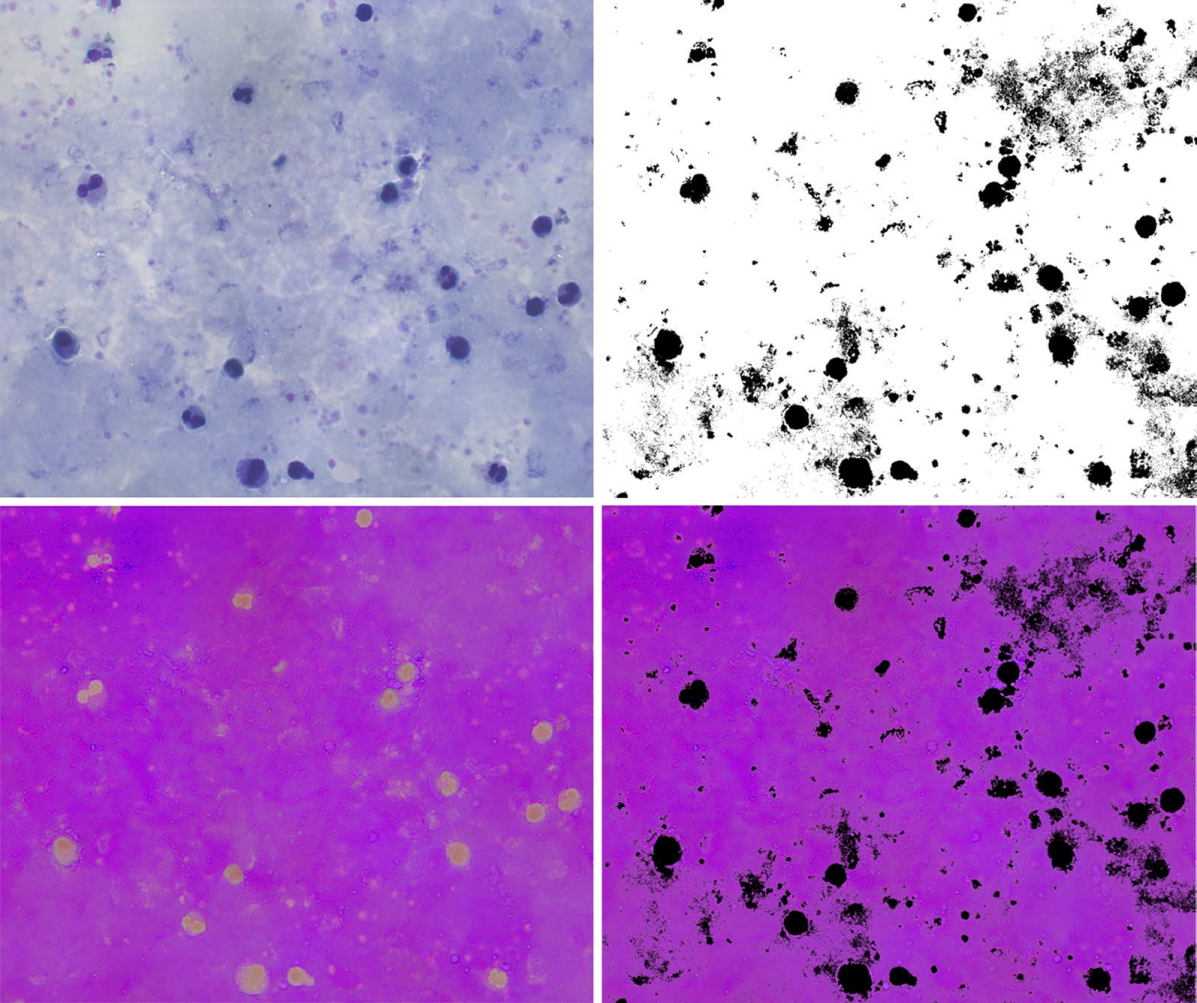
Background segmentation process. From left to right, the figure shows the original microscopic image in RGB, the image in the HSV colour space, the thresholded image (background information appears white and black), and the last image shows the background information in the HSV colour space used to analyze the coloration quality

Figure 3 shows examples of the images obtained in the different stages of the segmentation process. The first image corresponds to the original image in RGB. The second image corresponds to the image in the HSV color space. The third one is the image obtained after thresholding, where the background information appears in white and the foreground (leukocytes, parasites, and platelets) in black. Finally, the fourth image shows the original image in the HSV colour space, without the foreground information. This image is the one used to analyse the coloration quality. It is obtained after multiplying the thresholded image by the original one.

Different tests were conducted to identify the best strategy for segmenting the background of the smear. Section describes the results.

### Image-based criteria of quality: feature extraction

#### Exploration of colour spaces

After applying the thresholding process described above, the colour information of the resulting image (original image without foreground elements) was analysed. The analysis focused on finding differences between the information present in the background of images with good and bad coloration quality. The analysis was conducted using images of pattern thick blood smears from the INS-Colombia (used to evaluate diagnostic centers of the country), and images from field laboratories: five pattern images with good coloration quality; and 10 images from field laboratories, 5 with good and 5 with bad coloration qualities. For the analysis, the colour distribution of the HSV and GGB colour spaces (which are typically used by the research community for different malaria diagnosis stages [15, 17, 18, 29]) were used.

As a result, it was found that the histograms’ colour information distribution changes according to the coloration quality of the smear. The components H and S from the HSV colour space were the ones that better reflected a change in the colour distribution. The distribution of the data in the GGB colour space did not provide a way to differentiate the two background quality classes. Based on these results, the feature extraction process was focused on the HSV colour space.

#### Feature exploration

It was studied 19 different feature vectors based on variances, correlation coefficients, and histograms (specific variables from histograms, full histograms, and principal components from the histograms). The feature vectors were visually analysed to see their capability of classifying images into two classes: good and bad coloration quality.

- Histograms
  - Full histograms: The full histogram was analysed as a feature vector to test its utility as a coloration quality indicator in thick blood smears. It was extracted the individual H and S components from the HSV color space. Likewise, it was decided to combine the H and S components (HS) as a unique feature vector. This HS refers to the sum of the components H and S separately.
    It was also compared the differences between using the full histograms with 256 bins or only 16, in order to verify if the data distribution from the complete histogram can be maintained using less number of bins, as it has been reported in previous studies [9, 30]. In all the histograms, the first bin was removed because it corresponds to the foreground elements that were thresholded (black pixels). The V component from the HSV colour space was not used in the analysis because the V information does not appear to change with the coloration quality.
  - Specific variables from the histogram (H, S, V): A statistical discriminant analysis procedure was conducted to select from each component of the HSV colour space the best features that provide more information about the background coloration quality in TBS. The full histogram with 255 bins (after removing bin 1) was used. This analysis was done employing the statistical software SPSS$^{\text{\textregistered} }$ Statistics for Windows, Version 25.0, Armonk, NY: IBM Corp [31]. Details of the process are found in [32].
- Principal component analysis
  Principal-component analysis (PCA) is a technique used to reduce the dimensionality of multidimensional data. This process generates a new data set of variables named principal components [33]. The PCA was applied to the H, S and HS histograms (the ones with 15 bins), and the first 3 PCA components were extracted and selected for analysis. The PCA was applied using normalized and no-normalized data. Additionally, the feature vectors obtained were combined in one feature vector as another independent feature, as is presented in Table 2.
- Variances
  The variances of the H, S, and HS histograms (255 bins) were also analyzed as features. In this case, firstly, It was extracted the histograms values from the H, S, and HS combined components of the HSV color space as features vectors. It was employed the feature vector HS to use the combined H and S values in one variable. According to each one feature vector, the variances of the H, S, and HS histograms (255 bins) were also analysed as features. Table 2 shows the feature vectors created with these features.
- Correlation coefficients
  Correlation coefficients were obtained, comparing the images of the dataset with a reference image or image pattern. This image pattern was created using reference thick blood smears with good coloration quality, donated by the INS-Colombia, use as ground-truth to evaluate the protocols followed on field laboratories.
  From the 5 reference smears, a total of 50 images with good coloration quality were acquired (10 per smear). An average histogram from the pattern images was obtained ($\hbox {H}_{av}$, S$_{av}$, and $\hbox {HS}_{av}$), and correlation coefficients were calculated by comparing the average pattern histogram with the histogram of each image of the database. Table 2 shows the feature vectors created with these features.

**Table 2:** Summary of feature vectors

Feature vector	Type of data	Dimensions
Hist$_{15}$_H	Normalized	1 x 15
	No normalized	
Hist$_{15}$_S	Normalized	
	No normalized	
Hist$_{15}$_HS	Normalized	
	No normalized	
[$\hbox {H}_{5}$, $\hbox {S}_{35}$, $\hbox {V}_{114}$]	Normalized	1 x 3
	No normalized	
[$\hbox {H}_{PCA1}$, $\hbox {H}_{PCA2}$, $\hbox {H}_{PCA3}$]	Normalized	1 x 3
	No normalized	
[$\hbox {S}_{PCA1}$, $\hbox {S}_{PCA2}$, $\hbox {S}_{PCA3}$]	Normalized	
	No normalized	
[$\hbox {HS}_{PCA1}$, $\hbox {HS}_{PCA2}$, $\hbox {HS}_{PCA3}$]	Normalized	
	No normalized	
[$\hbox {H}_{PCA1}$, $\hbox {H}_{PCA2}$, $\hbox {H}_{PCA3}$, $\hbox {S}_{PCA1}$, $\hbox {S}_{PCA2}$, $\hbox {S}_{PCA3}$, $\hbox {HS}_{PCA1}$, $\hbox {HS}_{PCA2}$, $\hbox {HS}_{PCA3}$]	Normalized	1 x 9
	No normalized	
[$\hbox {H}_{var}$, $\hbox {S}_{var}$, $\hbox {HS}_{var}$]	Normalized	1 x 3
	No normalized	
[$\hbox {H}_{corr}$,S$_{corr}$,$\hbox {HS}_{corr}$]	No normalized	1 x 3

## Results

For selecting image criteria of quality for the background of thick blood smears, we followed a two-stage process presented in Fig. 1: (1) Background segmentation and (2)Feature extraction. This section presents experiments conducted to find and evaluate the best algorithm for background segmentation and the best feature extraction technique.

### Background segmentation

In this paper, the background colour information of microscopic TBS images is used to analyse the smear quality. To extract the background information from the foreground (platelets, parasites, but especially leukocytes), thresholding techniques were explored.

#### Threshold selection

An exploratory analysis with the HSV and RGB colour spaces was conducted to find the best threshold and the best colour space. The evaluation was based on the segmentation capability. The best colour space was carefully selected, ensuring that stained leukocytes were not confused as background information.

For this analysis, the National Reference Laboratory-LNR from INS-Colombia provided reference TBS, 5 of good coloration quality and 5 with bad coloration quality, both from field laboratories. It was captured one image per smear, which were used for the threshold selection and segmentation evaluation.

Besides, it was applied six transformations to the original images using the RGB and HSV colour spaces, and then the resulting image was thresholded. It was selected the best threshold value that best segments the background information from the foreground and can generalize for both scenarios, for TBS with good coloration quality and bad coloration quality. The six transformations were:

- RGB/Gray/Threshold: The RGB image was transformed to grayscale, and then thresholded.
- HSV/Gray/Threshold: The RGB image was transformed to the HSV colour space, then to grayscale, and finally thresholded.
- SV/Threshold: The RGB image was transformed into the HSV colour space. From this image, the pixels with an S and V values that fall under a specific threshold are considered to be background, otherwise foreground.
- H/Threshold: The RGB image was transformed into the HSV colour space. From this trasformation, only the H component was thresholded.
- S/Threshold: The RGB image was transformed into the HSV colour space. From this trasformation, only the S component was thresholded.
- V/Threshold: The RGB image was transformed into the HSV colour space. From this trasformation, only the V component was thresholded

The segmentation results were analysed visually. Table 3 shows the results of the thresholding process when applying different transformations to the original image. It was selected the best results according to their segmentation capacity. The components S and V combined (SV) were the components that showed the best segmentation results in contrast with the others (Table 3). Those components showed similar background segmentation capability for thick blood smears images of good and bad quality. On the contrary, the other colour spaces mixed with background information.

**Table 3:**
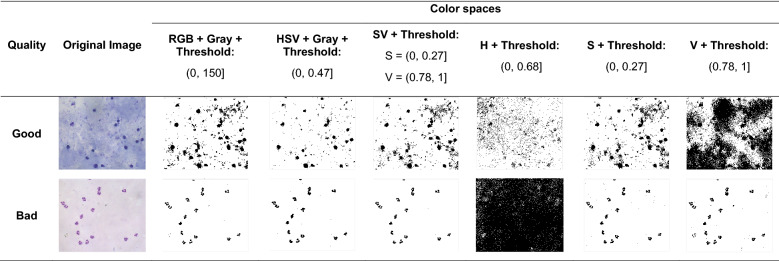
Threshold selection results. Background segmentation results from images of good (first row) and bad coloration quality (second row) using the RGB and HSV colour spaces, SV components, H component, S component and V component from HSV colour space. H was the component that showed the lower segmentation capability than the other components

#### Evaluation of Background Segmentation

To evaluate quantitatively the segmentation results obtained in the previous section, it was done a numerical verification of the number of segmented leukocytes. In each thresholded image obtained by each combination of colour spaces, the number of leukocytes were manually counted and compared to ground truth data (the total number of leukocytes present in the image). The true positive rate was calculated (see Table 4). As a result, the “SV + Threshold” strategy was the best one to extract the background information without mixing foreground information. This result corresponds to the one found with the visual evaluation of the images, conducted above.

**Table 4:** Background segmentation evaluation. Comparison of the number of leukocytes manually found in the thresholded image with the total number of leukocytes present in the image (ground-truth)

Name image	Quality	Ground truth	Segmented leukocytes number					
			RGB + Gray + Threshold	HSV + Gray + Threshold	SV + Threshold	H + Threshold	S + Threshold	V + Threshold
c1_4	Good	17	17	17	17	2	17	16
c2_3		31	31	29	31	0	31	31
c3_10		23	20	23	23	2	23	18
c4_4		26	26	26	26	18	26	19
c5_7		16	16	16	16	8	16	16
c51_10	Bad	14	14	14	14	5	14	14
c52_9		24	21	23	23	0	23	24
c53_10		15	15	15	15	6	15	15
c54_9		10	10	10	10	0	10	2
c55_5		6	6	6	6	5	6	6

### Feature extraction

Different feature vectors were selected, which are summarized in Table 2. To determine the best feature vectors, The Machine Learning Matlab [22] Toolbox was used to train 23 different classifiers available in the toolbox (Fig. 33). From this test it was selected the best four feature vectors and the best classifiers that can be used to automatically estimate the coloration quality from images of thick blood smears. The tests were conducted using the 420 images of the test set from the database.

For evaluating the results, the TNR (True Negative Rate), the TPR (True Positive Rate), and the F1-score were analysed. Nevertheless, the TNR was considered the most critical metric. This because of the implications of the results. A false-negative coloration quality analysis (predict good quality when the real coloration quality is bad) could have dangerous effects on patient health. The latter, because a smear that is not stained correctly can induce an error in the detection of the parasite, and therefore it may carry errors in the dosage calculation to patients, in addition to errors in the parasite density calculation.

From the 19 feature vectors that were tested, Table 5 presents the results of some of them. The first four rows show the feature vectors with the best results (>96%). The last two rows show the feature vectors that obtained the lowest rates (<91%) to use as a reference. The third column of the table shows the name of the classifier that obtained the best results. An interesting finding is that the best results (first four rows) correspond to feature vectors that include, in all the cases, the H component from HSV colour space, which is the component that contains the colour information.

**Table 5:** Summary of the features vector with higher rates

Feature vector	Dimensions	Classifier 1	TNR	TPR	F1-score
[$\hbox {H}_{PCA1}$, $\hbox {H}_{PCA2}$, $\hbox {H}_{PCA3}$]	1 x 3	Quadratic SVM	0,99	0,96	0,97
[$\hbox {H}_{corr}$, $\hbox {S}_{corr}$, $\hbox {HS}_{corr}$]	1 x 3	Cubic SMV	0,99	0,96	0,97
$\hbox {Hist}_{15}$_H	1 x 15	Quadratic SVM	0,99	0,97	0,97
[$\hbox {H}_{PCA1}$, $\hbox {H}_{PCA2}$, $\hbox {H}_{PCA3}$, $\hbox {S}_{PCA1}$, $\hbox {S}_{PCA2}$, $\hbox {S}_{PCA3}$, $\hbox {HS}_{PCA1}$, $\hbox {HS}_{PCA2}$, $\hbox {HS}_{PCA3}$]	1 x 9	Quadratic SVM	0,99	0,96	0,97
[$\hbox {S}_{PCA1}$, $\hbox {S}_{PCA2}$, $\hbox {S}_{PCA3}$]	1 x 3	Cubic SMV	0,91	0,81	0,85
[$\hbox {H}_{var}$, $\hbox {S}_{var}$, $\hbox {HS}_{var}$]	1 x 3	Ensemble (BUSBoosted trees)	0,88	0,87	0,87

Finally, the best feature vector was selected considering the distribution of the data in the feature space, but especially the classification rates. The best results were obtained with the 15-bins histogram of the Hue channel, the $\hbox {Hist}_{15-}$H. This feature vector was the one that allowed the separation between classes (Good/Bad coloration quality) with a $\hbox {TPR} =97\%$, $\hbox {TNR} = 99\%$ and $\text {F1-score} = 97\%$.

## Discussion

In the microscopic analysis of thick blood smears for malaria diagnosis, the back- ground is one of the most important features that allow declaring a determinant judgment about the coloration quality of the smear. With this in mind, in this paper, the background information in images of TBS was studied.

To conduct the study, it was created an image dataset where the illumination conditions were controlled during the acquisition process for allowing repeatability of the results and for guaranteeing that the appearance of the images correspond to the same ones perceived by the human eye trained for malaria diagnosis when the smear is visualized through the microscope. These are essential features of the dataset designed and conducted during this work, especially because several authors have mentioned that the absence of control during the image acquisition does not allow to make comparisons with the different published papers [9, 15, 17, 24, 25].

Image-based malaria diagnosis has been studied in the literature mainly focus on parasite detection and classification in thin blood smears [14–16, 18, 24, 28, 34]. The RGB and HSV colour spaces were the most common colour spaces for the segmentation of the parasite nucleus, erythrocytes segmentation, and even the background removal in images from thin blood smears. In [18] the HSV colour space was used to analyse images elements obtained from thick blood smears stained with the Jaswant Singh Battacharya (JSB) stain.

The type of stain used for the staining procedure of the smear can give different colour saturation to the biological elements. This work confirms that the HSV colour space is helpful for the analysis of components in images obtained from thick blood smears stained with the Romanowsky stain and likewise for the study of the background coloration quality.

This paper found that the best colour space for segmenting the background and extracting the feature vector was the HSV colour space. As a descriptor of the col- oration quality it was found that the 15 bins histogram of the Hue component from the HSV colour space was the one that provided better scores when classifying images of TBS between good and bad coloration quality.

Despite the importance of the coloration quality in thick blood smears, the image- based studies conducted until now have focused only on parasite detection and classification. The scientific society has forgotten to study the protocols that precede the blood sample analysis, such as sample taking, stain preparation, and sample staining, which impacts the acquisition of the images and, thus, the reliability of the diagnosis done for each patient. This work is a reference in the current literature that studies this essential facto, and it is the first to use computer vision to analyse the sample staining procedure of thick blood smears.

## Conclusions

It was analysed and present an image-based approach to describe the coloration quality of thick blood smears used for malaria diagnosis and stained with the Romanowsky stain. Estimation of the coloration quality is an important step for automating malaria diagnosis. The goal of automating malaria diagnosis is to support human vision improving diagnostic times. However, the absence of investigations where factors such as staining and slide preparation are controlled have made a fair automated-systems comparison difficult. For that reason, an image analysis system that allows the evaluation of the critical staining process of thick blood smears used in the microscopic diagnosis of *P. vivax* malaria is proposed and delivered.

This paper found that it is possible to find image criteria of coloration quality by extracting features in the HSV colour space. These features can be used to train an algorithm to classify the coloration quality of the images, obtaining good classification rates TNR and TPR > 95%.

In rural diagnostic centers, the developed algorithm could be beneficial to guarantee a good diagnosis despite errors in the staining procedure. The latter because by having in advance the estimation of the colouration quality can make microscopists aware that a careful analysis of each microscopic field is required. Additionally, this algorithm could be used for an objective evaluation of the abilities of microscopists, e.g. when evaluating microscopists’ smear staining capabilities.

## Data Availability

The dataset generated and analysed during the current study is available from the corresponding author on request.

## Abbreviations

WHO: World Health Organization
INS-Colombia: Instituto Nacional de Salud de Colombia (National Health Institute of Colombia)
LNR: Laboratorio Nacional de referencia (National Reference Laboratory)
SVM: Support Vector Machine
TNR: True Negative Rate
TPR: True Positive Rate
