# Correction to: Image features for quality analysis of thick blood smears employed in malaria diagnosis

**DOI:** 10.1186/s12936-022-04138-1

**Published:** 2022-04-25

**Authors:** W. M. Fong Amaris, Carol Martinez, Liliana J. Cortés-Cortés, Daniel R. Suárez

**Affiliations:** 1 Programa de Doutorado Em Biotecnologia, Universidade Federal Do Pará, Belém, Brazil; 2 School of Engineering, Pontificia Universidad Javeriana, Bogotá, Colombia; 3 Space Robotics (SpaceR) Research Group, Interdisciplinary Centre for Security, Reliability, and Trust (SnT), University of Luxembourg, 29, avenue John F. Kennedy, 1855 Luxembourg Kirchberg, Luxembourg; 4 Laboratory of Parasitology, National Health Institute of Colombia, Bogotá, Colombia

## Correction to: Malaria Journal (2022) 21:1–12 https://doi.org/10.1186/s12936-022-04064-2

Following publication of the article [1], the authors flagged that an incorrect version of affiliation 3 had been provided.

The affiliation has now been corrected in the original article and the updated affiliation can be seen in this correction.
